# Aortic Stenosis, a Left Ventricular Disease: Insights from Advanced Imaging

**DOI:** 10.1007/s11886-016-0753-6

**Published:** 2016-07-06

**Authors:** Sveeta Badiani, Jet van Zalen, Thomas A. Treibel, Sanjeev Bhattacharyya, James C. Moon, Guy Lloyd

**Affiliations:** Barts Heart Centre, St Bartholomew’s Hospital, West Smithfield, London, EC1A 7BE UK; Eastbourne District General Hospital, Kings Drive, Eastbourne, East Sussex BN21 2UD UK; Institute of Cardiovascular Science, University College London, London, EC1A 7BE UK; Institute of Advanced Cardiovascular Imaging, Queens Mary’s University of London, West Smithfield, London, EC1A 7BE UK

**Keywords:** Aortic stenosis, Left ventricle, Myocardial fibrosis, Echocardiography, Cardiac magnetic resonance

## Abstract

Aortic stenosis (AS) is the most common primary valve disorder in the elderly with an increasing prevalence. It is increasingly clear that it is also a disease of the left ventricle (LV) rather than purely the aortic valve. The transition from left ventricular hypertrophy to fibrosis results in the eventual adverse effects on systolic and diastolic function. Appropriate selection of patients for aortic valve intervention is crucial, and current guidelines recommend aortic valve replacement in severe AS with symptoms or in asymptomatic patients with left ventricular ejection fraction (LVEF) <50 %. LVEF is not a sensitive marker and there are other parameters used in multimodality imaging techniques, including longitudinal strain, exercise stress echo and cardiac MRI that may assist in detecting subclinical and subtle LV dysfunction. These findings offer potentially better ways to evaluate patients, time surgery, predict recovery and potentially offer targets for specific therapies. This article outlines the pathophysiology behind the LV response to aortic stenosis and the role of advanced multimodality imaging in describing it.

## Introduction

Aortic stenosis (AS) is the most common left-sided valve lesion [1], and moderate or severe AS affects more than one in eight people over 75 [2]. Current indications for aortic valve replacement are based around the severity of stenosis, or in asymptomatic patients with evidence for left ventricular compromise (ejection fraction (LVEF) < 50 %). Aortic intervention is also indicated for those who exhibit symptoms during exercise or who are undergoing cardiac surgery for other indications [3••, 4]. Historically, the majority of patients with severe AS receiving aortic valve replacement exhibited a high transvalvular gradient, with variable left ventricular hypertrophy and normal or reduced LVEF. Populations with lower gradient yet still severe aortic stenosis are increasingly recognised in situations of low flow either characterised by an impaired ejection fraction (classical low flow-low gradient aortic stenosis) or a normal ejection fraction (paradoxical low flow-low gradient aortic stenosis) [5]. The previous assumption has been that patients progress from normal systolic function and high gradients through to impaired function and low flow, hence low gradients. This is now considered to be at best an oversimplification and at worst erroneous as the development of these ultimate severe AS phenotypes appears to follow different antecedent pathological pathways [6]. This illustrates that this disease is highly dependent on the responses of the myocardium and vasculature [7–9]. This response is complex and consists of a combination of wall thickening, change in cavity size and fibrosis, with the associated effects on systolic and diastolic function [10]. This is further complicated by comorbidity associated with progressive ageing, in particular coronary artery disease, hypertension, diabetes and the progressive decreases the compliance of the vascular bed.

## Classical Aortic Stenosis

In classical aortic stenosis, a longstanding increase in global LV afterload results in concentric LV hypertrophy [11] and an elevation of end-diastolic pressure [12]. This hypertrophy is the main compensatory mechanism in the early phase of the disease, limiting wall stress and maintaining normal endocardial shortening [13] by offsetting the raised intracavity pressure [10]. Cardiac output and LV filling pressures may remain within normal limits despite very high pressures. This is characterised by a slow increase in transvalvular gradient until finally flow drops and the gradient start to reduce. The changes in the extracellular collagen matrix and a decrease in myocardial perfusion pressure eventually lead to subendocardial ischaemia [14], resulting in myocardial apoptosis and fibrosis [11]. In addition, many factors including age [15], sex [16], genetic factors, hypertension and the presence of coronary artery disease influence ventricular behaviour and remodelling. The additive effect of concomitant systemic hypertension leads to a significant increase in left ventricular systolic wall stress, which further increases the global afterload. The result is further impairment of myocardial function leading to reduced survival [17] with increased perioperative complications and mortality [18, 19].

## Non-classical Aortic Stenosis Progression

This description with the progression of severe aortic stenosis through compensatory mechanisms resulting in a high gradient followed by left ventricular failure resulting in a reduction in gradient is attractive but unfortunately does not describe the majority of patients destined to develop severe AS with a low transvalvular gradient, whether or not the ejection fraction is preserved or impaired. A proportion of such patients demonstrate excessive left ventricular hypertrophy [20]. Dahl et al. demonstrated that in patients with LFLGAS (low flow, low gradient aortic stenosis), only 5 % had evidence of previous high gradient disease. In these patients with LFLGAS, there was a gradual increase in wall thickness, reduction in end diastolic diameter and small reduction in EF, over a 5-year follow-up period. The results suggest that LFLG AS with preserved LVEF is not an end-stage high-gradient disease but a separate entity characterised in part by progressive maladaptive LV remodelling [21]. The pathology that defines which way the disease will request remains to be fully described.

## Factors Affecting the Left Ventricular Response to AS

When the limit of sarcomere extension and diastolic stiffness is reached, the “adaptive” mechanisms to pressure overload are exceeded. The left ventricle becomes unable to maintain a normal stroke volume in the setting of limited preload reserve, a condition known as “afterload mismatch”. The afterload mismatch state definition, however, implies that myocardial contractility is not depressed and that LV size and function will recover, once the valvular obstruction is relieved [22]. A potential underlying mechanism involving the transition from left ventricular hypertrophy/high afterload states to heart failure is the increase in LV filling pressure leading to increased left atrial wall tension and myocyte stretch. In imaging, this is observed by deterioration in diastolic properties that improve after correction of the obstruction [23] which is closely related to clinical outcome [24]. The magnitude and chronicity of the increased LV filling pressure are associated with an increase in left atrial size [25], which has been shown to predict postoperative symptomatic improvement [26] and subsequent prognosis [27]. This process results in fibrosis, apoptosis and, in turn, atrial enlargement [28]. The amount of myocardial fibrosis and myocardial degeneration affects systolic and diastolic function [11, 29] and appears to have a significant effect on clinical status and long-term survival after aortic valve replacement [30], suggesting at least partially irreversible remodelling.

## Patterns of Remodelling

The ventricular hypertrophic adaptation follows four well-recognised mechanistic patterns: normal ventricular geometry, concentric remodelling, concentric hypertrophy and eccentric hypertrophy [31]. Concentric remodelling is defined by a normal left ventricular mass and an increased relative wall thickness and concentric hypertrophy by a combination of left ventricular hypertrophy and increased relative wall thickness [10]. There is evidence to suggest that adaptive remodelling becomes maladaptive with increasing LV hypertrophy and consequent myocardial fibrosis. A study by Cioffi et al. showed that over 10 % of patients with asymptomatic severe AS exhibited inappropriate left ventricular hypertrophy (LVH) and these patients had a 4.5-fold higher risk of death, aortic valve replacement (AVR) or hospital admission [20]. Echocardiographic studies, however, have also demonstrated that 10–20 % of patients with aortic stenosis do not have left ventricular hypertrophy [32, 33]. The severity of AS has been shown to be unrelated to the pattern of hypertrophy, supporting the multifactorial nature of LV remodelling [34]. In LFLGAS, longitudinal myocardial shortening is affected to a larger extent due to a more advanced fibrosis in the subendocardial layer, where the fibres are oriented longitudinally [35, 36]. It is associated with concomitant systemic hypertension in around 77 % of patients [37], which may induce a low flow state despite normal LV ejection fraction. The LV therefore faces a double afterload due to the valvular load due to the AS and an arterial load, as a consequence of reduced arterial compliance. Paradoxical (preserved EF) low flow low gradient AS is characterised by a restrictive physiology, more pronounced LV remodelling and myocardial fibrosis contributing to a reduction in size, compliance and filling of the LV [5, 38]. The advanced fibrosis in the subendocardial layer is associated with a reduction in longitudinal shortening. These patients have a worse prognosis than those with moderate AS or normal flow severe AS [17, 39]. The prognosis of these patients is also worse if treated medically rather than surgically. A study by Barash found a 2-fold increase in mortality and an almost 50 % lower referral rate for AVR in the low gradient AS compared to the high gradient AS group [40]. Hachicha et al. studied the data of 512 patients with severe aortic stenosis and found that compared with normal flow aortic stenosis, the overall 3-year survival was lower in patients with low flow AS [17].

## The Role of Advanced Imaging

### Defining Severe AS

For the purposes of patient care severe, AS is usually defined as a calculated aortic valve area of <1.0 cm^2^ (or <0.65/m^2^) corroborated by a peak aortic jet velocity of >4.0 m/s and a mean transvalvular pressure gradient of >40 mmHg [3••, 4]. The classification of stenotic severity is not always so straightforward, and the echocardiographic findings are discordant in one in three patients [41, 42], the most frequent being a valve area in the severe range with a low mean gradient <40 mmHg, suggesting a lesser severity of AS. Here, a distinction needs to be made between those with low flow (conventionally considered <35 mls/m^2^) and frequently low ejection fraction, and those with normal flow, in whom measurement inaccuracy or the inherent discordance between a mean gradient of 40 mmHg and a valve area of 1.0 cm^2^, are the more likely explanations. Ensuring the correct definition of severe AS remains an important challenge both clinically and also in critical evaluation of the literature [43].

### Transthoracic Echocardiography

Transthoracic echocardiographic assessment includes measures of AS severity, LV function and LV wall thickness, and cavity dimensions and ejection fraction should be based on existing recommendations [44••, 45••]. Parameters describing aortic stenosis severity include transvalvular velocity and mean gradient measured from continuous wave Doppler through the left ventricular outflow tract and aortic valve. Particular care should be made to interrogate this signal from multiple positions on the left and right side of the chest: Up to 61 % of patients do not have their highest signal from the conventional apical position [46]. Both of the measurements are heavily flow dependent, and a variety of approaches are employed to correct for this. The principle among these is the use of the continuity equation to correct for sub-aortic flow. This provides the calculation for the aortic valve area. It requires a measurement of the left ventricular outflow tract (LVOT) areas estimated from its diameter using the equation [47]. This may be a major source of error as small errors are squared; furthermore, the LVOT is usually elliptical in severe AS [48, 49]. The dimensionless index where the ratio of the sub-aortic and transaortic flow is described attempts to circumnavigate this [50, 51]. Other measures such as stroke work loss have been shown to obviate the assessment of transvalvular flow and appear to be more stable than the Gorlin valve area in the assessment of stenotic valve lesions [52–54], and measurement of valvular resistance (the ratio of transvalvular gradient to flow) has also been proposed as a valid method for quantification of aortic stenosis severity [55, 56]. A further Doppler parameter, the energy loss index (ELI) has been shown to provide independent and incremental prognostic information than that derived from the conventional markers of aortic stenosis severity, in asymptomatic patients with aortic stenosis [57].

Decreasing flow may be due to an increase in global LV afterload not only due to the valvular stenosis but also from a decrease in systemic arterial compliance and/or increased vascular resistance [58, 59]. Assessment of the global LV haemodynamic load by measuring the valvuloarterial impedance (Zva) was proposed by Pibarot and co-workers [60] and is defined as the ratio of the estimated LV systolic pressure (i.e. the sum of the systolic arterial pressure (SAP) and mean pressure gradient (MPG) to the stroke volume indexed (SVi) for body surface area [61]. The Zva has been shown to be superior to the standard parameters of AS severity such as transvalvular gradients and effective orifice area in predicting LV dysfunction and clinical outcomes [27, 62, 63].

Dichotomising function using ejection fraction is a major oversimplification as those with small cavity size (due to either habitus or hypertrophy), or significantly impaired long axis function may also develop low flow. Deformation imaging (strain and strain rate) using speckle-tracking echocardiography has been shown to be more sensitive than LVEF in detecting myocardial contractility [36, 64]. Two-dimensional speckle tracking echocardiography (2D-STE) shows the deformation of the left ventricular muscle in three directions: longitudinal, circumferential and radial. Studies in patients with severe AS and preserved LVEF confirm that the decrease in LV longitudinal strain [65, 66] predicts events in asymptomatic patients, as well as mortality [67]. Impaired strain prior to valve replacement predicts worse postoperative outcome with respect to rehospitalisation for heart failure and overall mortality [68]. Global longitudinal strain (GLS) appears to be a more robust parameter in the assessment of subclinical LV dysfunction. GLS is decreased in AS patients and is even more affected in severe AS patients as compared to patients with moderate AS [69, 70]. Patients with LV longitudinal strain ≤15.9 % have an excess risk of death, symptoms or surgery that was more than twice that of patients with preserved longitudinal function [71]. In asymptomatic patients with severe AS and preserved LV ejection fraction, a significant decrease in LV longitudinal strain (especially in the basal segments) signals a reduced exercise capacity and an increased risk of cardiac events was observed during follow-up in patients with lower values of longitudinal strain in the LV basal segments (below −13 %), while a GLS below −18 % predicted an abnormal exercise response with a sensitivity of 68 % and a specificity of 77 % [72].

Further information can be gained by increasing flow through the valve using low-dose dobutamine stress echo (DSE) (class IIa indication in managing patients with a reduced ejection fraction) [3••, 4]. It is likely that it offers information in those with preserved EF, but this is less well established. The assessment of LV flow reserve during low-dose DSE has clear prognostic implications in true severe AS [73, 74]. If a 20 % increase in stroke volume is associated with the development of a gradient of 40 mmHg or a peak velocity of greater than 4 m/s, with an unchanging valve area, then the AS is severe and intervention is warranted, while if no flow recruitment is observed then the outlook from surgery is very poor. Dobutamine stress echo has a critical role in distinguishing between “true severe” AS (due to the valve itself) and pseudosevere AS, which is predominantly due to myocardial disease. Pseudosevere AS will demonstrate an increase in EOA but relatively little increase in gradient [5]. Those with pseudo severe disease are less likely to show a favourable response to intervention. The TOPAS study investigators proposed to calculate the projected (effective orifice area) EOA that would have occurred at a standardised flow rate of 250 ml/s, and this new parameter of 1.2 cm^2^ has been shown to be more closely related to actual AS severity, impairment of myocardial blood flow, LV flow reserve and survival than traditional DSE parameters [75, 76].

Myocardial response during exercise may also be useful in patients with severe AS who claim to be asymptomatic or who have equivocal symptoms [11], as is the assessment of exercise-induced changes in LV systolic function, which can provide prognostic information in patients with severe AS. The absence of LV contractile reserve is characterised by the absence or only small increase in LVEF and is associated with exercise-induced symptoms and a markedly reduced midterm cardiac event-free survival [77].

### CMR

Two predominant types of myocardial fibrosis mediate the transition from the initially adaptive hypertrophic response to decompensation to heart failure. Diffuse or interstitial fibrosis reflects the more uniform nature of the condition, whereas replacement fibrosis occurs later as the disease advances and is characterised by a more focal distribution [78, 79]. Myocardial biopsy remains the gold standard for validating myocardial fibrosis but is invasive, susceptible to sampling errors and unable to assess the fibrotic burden of the whole heart. Cardiovascular magnetic resonance (CMR) is the only non-invasive imaging modality that offers a direct, whole heart assessment of myocardial fibrosis [80]. Late gadolinium enhancement (LGE), for direct visualisation and quantification of focal replacement fibrosis, and T1 mapping, for assessing more diffuse patterns of interstitial fibrosis, are the two approaches commonly used in this context [81].

LGE CMR is the most accurate way to visualise focal midwall myocardial fibrosis [82, 83], which has been demonstrated in 19 to 62 % of patients with aortic stenosis [84, 85]. It is mainly found in the subendocardial layer of the LV, and its degree decreases from the base to the apex [86]. Its presence is associated with adverse postoperative outcomes, in particular not only residual symptoms but also mortality in patients undergoing valve replacement [87, 88]. Lee et al. carried out a prospective study of 118 patients with moderate and severe AS. They found that patients with left ventricular systolic dysfunction and LGE on CMR showed adverse structural and functional remodelling, and in patients with normal LV ejection fraction, LGE was associated with a stiffer ventricle, suggesting that LGE CMR may be useful in detecting subclinical LV dysfunction in these patients [89]. A smaller study carried out by Park et al. showed that the presence of LGE had effects on poor improvement of LV filling pressure in patients with severe AS. They also found that echocardiographic parameters such as tissue Doppler imaging (TDI) *E*′ were associated with the presence of LGE, suggesting that they may play a role in predicting long-term outcome and improvement in LV remodelling after AVR [77]. The degree of LGE has also been shown to correlate well with the degree of histological fibrosis in these patients [90].

LGE relies on a difference in signal intensity between normal and focal regions of myocardial fibrosis and is therefore not optimal for assessing interstitial fibrosis [91]. Diffuse interstitial fibrosis is more uniformly distributed than focal replacement fibrosis and is emerging as a potential treatment target, due to its reversibility. T1 mapping has emerged as a novel CMR technique to assess this form of fibrosis [92], by improving myocardial characterisation through its ability to quantify signal intensity for each voxel in the myocardium [93]. Several T1 mapping approaches have been developed to quantify diffuse fibrosis. Extracellular volume fraction (ECV), which corrects for blood pool and the plasma gadolinium volume of distribution, offers the best reproducibility and can predict outcomes as least as strongly as LV ejection fraction [94, 95].

T1 mapping has been studied in patients with aortic stenosis, demonstrating the presence of diffuse fibrosis, severity of aortic stenosis, LV mass and cardiac performance. A study by Bull et al. showed increased T1 values in patients with severe AS, with correlation with fibrosis on histology. Symptomatic patients were more likely to demonstrate increased T1 values compared to asymptomatic patients [81]. The degree of myocardial fibrosis has also been shown to correlate with symptoms and LV function. Patients with severe fibrosis were less likely to show improvement in symptoms, LV function and LVH after surgery compared with those patients with mild to moderate fibrosis [30]. A study by Treibel et al. used T1 mapping to investigate and correlate macroscopic and tissue level patterns of LV remodelling in patients with severe AS (NCT). They found that patients have differing patterns of remodelling, with both native T1 and ECV correlating with prognostic markers such as NT-pro-BNP [94].

The measurement of diffuse fibrosis has the potential to improve therapeutic management in patients with aortic stenosis, which may develop without symptoms or changes in LV function. A subgroup of patients with severe AS benefiting from early intervention may be identified.

### Coexistent Amyloid in Aortic Stenosis

Recent case reports and case series have reported coexisting cardiac amyloid in patients with severe aortic stenosis—typically wild-type transthyretin (wtATTR) [95–97]. Its prevalence and prognostic significance are currently unknown but require further investigation because they may influence the management of AS in terms of decisions surrounding intervention, procedure performance and the use of specific amyloid therapies.

### Computed Tomography

Cardiac computed tomography is heavily used in the clinical work-up of patients undergoing transcatheter aortic valve implantation (TAVI) in the assessment of aortic root, the thoracic aorta and vascular access [98]. There is a strong linear relationship between the extent of calcification and severity of aortic stenosis, which is often ignored in general CT chest reporting [99]. This has been shown to predict the progression of the condition and is particularly useful when ventricular function is reduced and stress echocardiography is ambiguous [100]. It is increasingly likely that calcification quantified by CT will become a better gold standard for aortic stenosis severity as it correlates very strongly with aortic weight [101]. Inflammation and calcification of the aortic valve are believed to play a key role in predicting disease progression. Work using 18F-NaF and 18F-FDG shows that activity of both tracers is increased in patients with both aortic sclerosis and stenosis, with a progressive rise in uptake with increasing disease severity [102].

With regard to the myocardium, the assessment of LV function and scar imaging by hyper enhancement post-iodine contrast with cardiac CT is not routinely established in clinical practice due to a high radiation dose, but constant technical advances may make this modality more attractive in the future. Finally, in parallel with the CMR T1 mapping technique, ECV can be calculated using equilibrium contrast CT and may be an attractive addition in patients not able to undergo CMR [103].

**Fig. 1:**
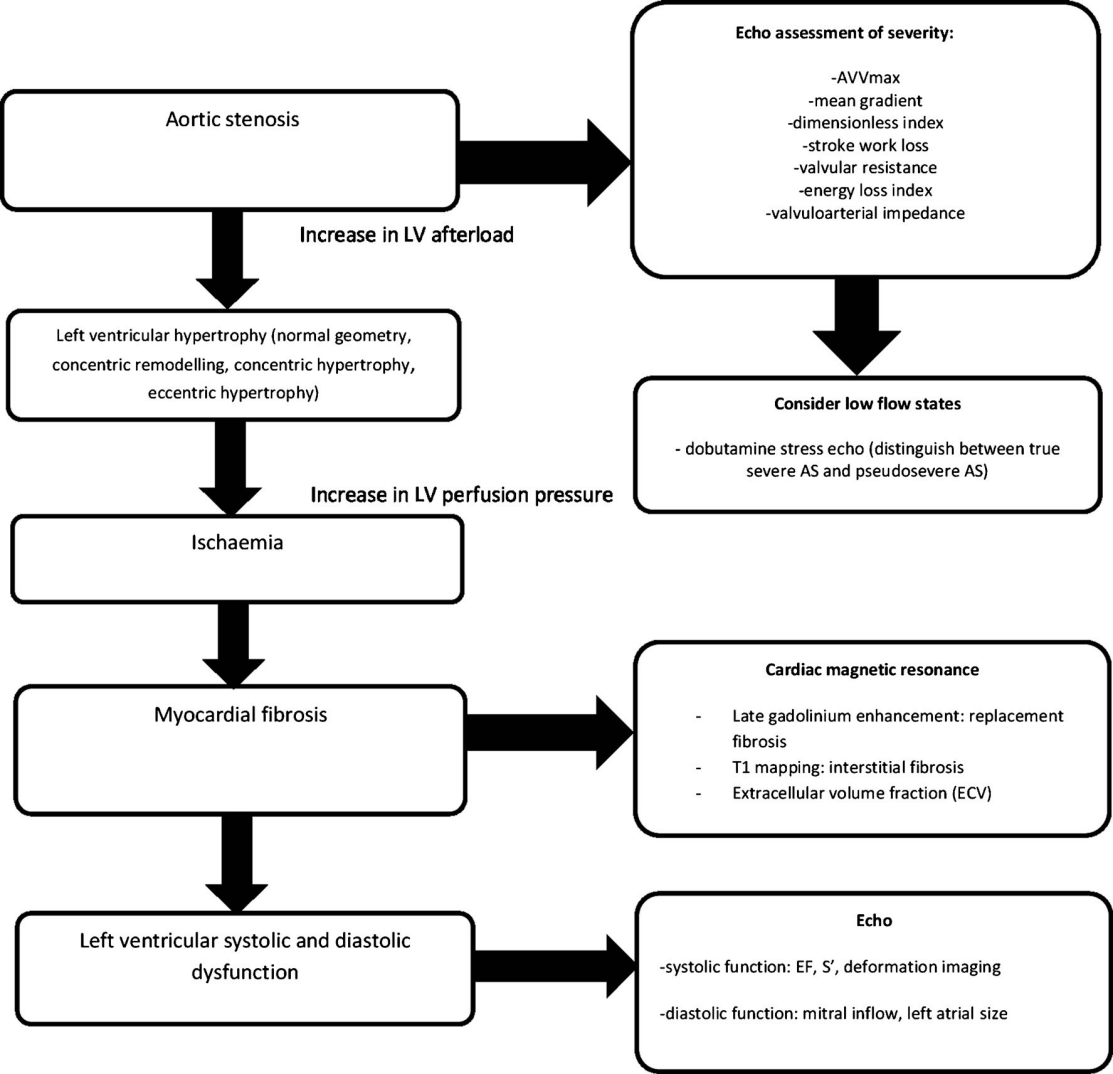
Flow diagram illustrating the left ventricular effects of aortic stenosis and the role of advanced imaging modalities in their evaluation

A flow diagram describing the different patterns of ventricular remodelling in aortic stenosis and the role of advanced imaging is outlined in Fig. 1.

## Conclusions

Aortic stenosis is a common lesion but with a complex phenotype, which can make assessment challenging in those patients with results that are discordant and/or flow is reduced. The assessment of left ventricular function by means of LVEF measurement is not adequate, and more detailed evaluation of myocardial mechanics, myocardial remodelling and flow is required to understand the pathology and assess the likely benefit to be gained from aortic valve intervention. Stress echocardiography, cardiac MRI and PET/CT all provide diagnostic and prognostic information, and an integrated multimodality approach to evaluation will identify those patients likely to benefit from aortic valve intervention when the clinical scenario is not clear cut. Follow-up for those with moderate aortic stenosis needs to be more rigorous as the previous assumptions that these patients would progress through the stage of high gradient before left ventricular is erroneous.

## Abbreviations

AS: Aortic stenosis
LV: Left ventricle
LVEF: Left ventricular ejection fraction
PLFLGAS: Paradoxical low flow-low gradient aortic stenosis
LFLGAS: Low flow low gradient aortic stenosis
AVR: Aortic valve replacement
LVOT: Left ventricular outflow tract
ELI: Energy loss index
SAP: Systolic arterial pressure
MPG: Mean pressure gradient
Svi: Stroke volume index
GLS: Global longitudinal strain
DSE: Dobutamine stress echocardiography
EOA: Effective orifice area
CMR: Cardiovascular magnetic resonance
LGE: Late gadolinium enhancement
TDI: Tissue Doppler imaging
ECV: Extracellular volume
NT-pro-BNP: N terminal prohormone of brain natriuretic peptide
wtATTR: Wild-type transthyretin
TAVI: Transcatheter aortic valve implantation
